# Quantifying social capital creation in post‐disaster recovery aid in Indonesia: methodological innovation by an AI‐based language model

**DOI:** 10.1111/disa.12631

**Published:** 2024-06-11

**Authors:** Daniel Moritz Marutschke, Muhammad Riza Nurdin, Miwa Hirono

**Affiliations:** ^1^ Faculty of Economics and Business Administration Kyoto University of Advanced Science Japan; ^2^ Asia Japan Research Institute, Ritsumeikan University Japan; ^3^ College of Global Liberal Arts, Ritsumeikan University Japan

**Keywords:** artificial intelligence (AI), disaster recovery, disaster response, language model, local community, machine learning, mixed methods, natural language processing, social capital, word embeddings

## Abstract

Smooth interaction with a disaster‐affected community can create and strengthen its social capital, leading to greater effectiveness in the provision of successful post‐disaster recovery aid. To understand the relationship between the types of interaction, the strength of social capital generated, and the provision of successful post‐disaster recovery aid, intricate ethnographic qualitative research is required, but it is likely to remain illustrative because it is based, at least to some degree, on the researcher's intuition. This paper thus offers an innovative research method employing a quantitative artificial intelligence (AI)‐based language model, which allows researchers to re‐examine data, thereby validating the findings of the qualitative research, and to glean additional insights that might otherwise have been missed. This paper argues that well‐connected personnel and religiously‐based communal activities help to enhance social capital by bonding within a community and linking to outside agencies and that mixed methods, based on the AI‐based language model, effectively strengthen text‐based qualitative research.

## 要旨

災害の影響を受けたコミュニティとの円滑な交流は、コミュニティの社会資本を構築および強化することができ、災害後の復興支援をより効果的に提供することにつながる。相互作用の種類、生成される社会資本の強さ、および成功した災害復興支援の提供の関係を理解するには、複雑な民族誌的かつ質的研究が必要であるが、それは少なくともある程度は研究者の直感に基づいているため、例示にとどまる可能性が高い。このような研究に次元を加えて強化するために、この論文では、定量的な AI ベースの言語モデルを使用した革新的研究手法を提示する。本モデルを使用することで、質的研究のデータを再調査して結果を検証し、見逃されていた可能性のあるその他の洞察の収集が可能となる。本論文では、人間関係の良好な人材と宗教に基づいた共同体活動が、コミュニティ内での絆や外部機関とのつながりによって社会資本の強化に役立つと論じている。また、AI ベースの言語モデルに基づく混合手法により、テキストベースの質的研究を効果的に強化できると論じている。

## 摘要

与受灾社区的顺畅互动可创造和加强社区的社会资本，从而更有效地提供灾后恢复援助。为了理解互动类型所产生的社会资本强度以及成功的灾后恢复援助之间的关系，需要进行复杂的民族志定性研究，但它可能仍具有说明性，因为它至少在某种程度上基于研究人员的直觉。为了增加维度并加强此类研究，本文提出了一种采用基于定量人工智能的语言模型的创新研究方法。该模型允许研究人员重新审查数据，从而验证定性研究的结果，并收集定性研究可能错过的额外见解。本文认为，人脉广泛的人员和以基于宗教的社区活动有助于通过社区内的联系和与外部机构的联系来增强社会资本。它还认为，基于人工智能语言模型的混合方法能有效地加强基于文本的定性研究。

## 1 INTRODUCTION: THE NEED FOR MIXED METHODS

How does local knowledge save lives and make post‐disaster recovery aid more effective? One answer to this question may lie in the formation and reinforcement of social capital. In a way to financial capital defined as ‘wealth or property that … can be invested or used to start a business’ (Oxford Learner's Dictionaries, 2024), social capital can be defined as ‘normative wealth’ in the form of trust, reciprocity, and normative values to start and engage in a relationship—often underpinned by local knowledge and negotiated between the members of a community or communities.

Increased trust, reciprocal relationships, and the sharing of normative values within and between communities matter hugely when it comes to the effectiveness of post‐disaster recovery aid, as illustrated in the paper by Muhammad Riza Nurdin (MRN) in this special issue of *Disasters*. Focusing on cases in two provinces in Indonesia, East Java and Aceh, the paper demonstrates the critical importance of the role of social capital in locally‐led post‐disaster recovery aid by showing how it is formed and enhanced, through ‘bonding’ within a community, ‘bridging’ between communities, and ‘linking’ a community to resources and goodwill outside of the community. MRN's ethnographic research deliberately uses a qualitative approach to capture the process of social capital formation by analysing community members' perceptions, collected through interviews and participant observation.

Taking a qualitative approach to social capital formation is rather rare, but Svendsen (2006, p. 65) has long advocated its importance because social capital is being built ‘at a micro level and *in situ*, i.e., in a specific time‐space context’. This runs counter to the majority of social capital research in the literature, which aims to ‘measure’ social capital using quantitative methods. For example, many studies carry out surveys to measure social capital by asking respondents about their level of participation in a local community, the degree of neighbourhood associations, connections between family and friends and those at work, levels of proactivity in social contexts, feelings of trust and safety, tolerance of diversity, and perceptions on the value of life (Bullen and Onyx, 1998). However, if we accept that social capital formation is ‘inseparably related to concrete discourses, social classifications, and identities in specific time‐space contexts’ (Svendsen, 2006, p. 42), then qualitative research that underpins such discourses, classifications, and contexts is imperative in creating a nuanced understanding of the fabric of effective post‐disaster recovery aid.

Qualitative research, though, tends to remain illustrative. Its generalisability is questionable because it is often based on a researcher's intuition or insight—although we do not intend to overlook the importance of such intuition and insight for ethnographic research. Our claim is that a quantitative approach is also needed to validate and confirm the findings of the qualitative research, and to advance research in general by offering more detailed and systematic analysis or by bringing some previously unnoticed elements into the academic spotlight. Local knowledge exists in people's minds in a particular setting. Therefore, it is important to have a robust methodology that can identify the process of social capital formation and, by extension, identify ‘local knowledge’ as objectively as possible.

This paper thus represents a step towards methodological innovation in the study of social capital formation in the context of responding to a disaster. Rather than resorting to the traditional survey‐based quantitative approach, the paper develops an artificial intelligence (AI)‐based language model to analyse data, consisting of interview transcripts and fieldnotes arising from participant observation, collected by MRN in his two Indonesian case studies: one relating to the Mount Kelud volcanic eruptions in East Java in 2014; and the other to the earthquake in the Gayo Highlands in Aceh in 2013. By deliberately using the same set of data, the paper aims to compare the results of MRN's qualitative content analysis with the results of a quantitative analysis using the proposed AI‐based language model.

The paper, therefore, asks the same research question as that posed in MRN's paper:

1. How do faith‐based organisations (FBOs) conduct their post‐disaster recovery programmes through social capital formation?^1^
  It also introduces two further questions, which focus more specifically on validating the findings of MRN's research results, thus ascertaining the validity of this novel mixed‐methods research approach:
2. Can the results of MRN's qualitative paper be confirmed through quantitative analysis using the AI‐based language model? And if so, how? In other words, can we use so‐called word embeddings to analyse the interview data specifically in relation to the process of social capital formation?
3. What new insights does the approach using the AI‐based language model provide?

This paper begins with Putnam's (1995, p. 67) often used definition of social capital as ‘features of social organization such as networks, norms, and social trust that facilitate coordination and cooperation for mutual benefit’. The paper takes a two‐tiered approach to the operational definition of social capital. The first tier is about the *ends* of building social capital. The ‘features of social organization’ mentioned by Putnam reflect three types of social capital:

- ‘bonding’ within a community, which centres on horizontal ties between community members who are primarily homogenous (Aldrich, 2012);
- ‘bridging’ between communities, which brings together people from neighbouring communities, or people from across social divisions within a community, and reduces barriers to collective action and speeds up their recovery (Szreter and Woolcock, 2004; Panday et al., 2021); and
- ‘linking’ with a group with ‘explicit, formal or institutionalized power or authority gradients in society’ (Szreter and Woolcock, 2003, p. 655).

The second tier of social capital, by contrast, amounts to the *means* by which actors (FBOs in the context of this paper) build social capital, for example, sharing events, common activities, history and culture, and human resources. The *ultimate* ‘ends’ in building social capital are to deliver post‐disaster recovery assistance successfully, but they are beyond the scope of this paper. The analysis here focuses on the means and *immediate* ends of social capital formation.

The rest of the paper is divided into three sections. The first discusses how the mixed‐methods approach of combining an AI‐based language model and qualitative ethnographic research advances our methodology by enabling researchers to engage in more robust analysis. It begins by comparing the traditional statistical language model with the contemporary neural network model (which provides the basis for the AI‐based language model), and then discusses the main features of the AI‐based language model. The second section outlines how we implement the AI‐based language‐model approach for our analysis. The third section presents the results of the AI‐based language model method and compares them with the results of the qualitative method.

The paper concludes by arguing that the AI‐based language model shows that FBOs conduct their post‐disaster recovery programmes by enhancing social capital, through well‐connected personnel and religiously‐based communal activities. This is almost the same research result as the one deriving from MRN's qualitative research. One difference is that the AI‐based language model sheds light on the importance of other types of personnel, not simply on the village facilitators alone. Methodologically, the paper argues that the AI‐based language model allows researchers to re‐examine interview data and thus validate the findings of the qualitative research. The model further gleans additional insights that the qualitative research might otherwise have missed. Using an AI‐based language model could therefore be beneficial to other research on the importance of local knowledge for effective post‐disaster recovery aid, because of its ability to conduct in‐depth *contextual* analysis *quantitatively*. This enhances the accuracy and depth of contextualisation by reflecting greater complexity in the data.

## 2 WHY USE AN AI‐BASED LANGUAGE MODEL?

The reason for this paper's focus on the utilisation of an AI‐based language model is that doing so permits researchers to examine the meaning of each word used in interview transcripts and fieldnotes in a context‐specific way. When considering the nature of local knowledge in relation to post‐disaster recovery aid—which is the main theme of this special issue of *Disasters*—the key concern is that knowledge is inherently contextual. Whether we take a qualitative or a quantitative approach, it is important to analyse community members' knowledge and perceptions by paying full attention to the context in which they spend their ‘everyday life’. The importance of the context is emphasised in ethnography, but the AI‐based language model allows researchers to understand it more systematically.

While the traditional statistical language model enables researchers to assess how many times a particular word appears as compared to other words, it is unable to retain the ‘rich information’ that attaches to each word, which enables one to comprehend the context in which each word is mentioned. ‘Rich information’ refers to the way the particular word relates to other words, and the degree of relatedness can be measured by a ‘similarity score’. The AI‐based language model is ‘trained’ on a specific corpus of texts (which can include any number of documents) in such a manner that it can then study all of the data inputs and analyse the relationships between each individual word and all of the other words in the corpus. This provides the rich information that allows one to understand the context of each word, necessary to computing the similarity score.

For instance, each time the word ‘earthquake’ is encountered, the words before and after it are inspected and the relationship with these words is updated. As we discuss further in the results section, the word ‘earthquake’ (*gempa* in Bahasa Indonesia) has a similarity score with other words as follows (in descending order):

**Table:** 

2013 (2013)	0.876
Bener Meriah (*meriah*)	0.861
Yogyakarta (*jogya/jogja*)	0.838/0.809
history (*sejarah*)	0.837
conflict (*konflik*)	0.832
Hendra (*henra*)	0.828
Bantul (*bantul*)	0.827
Langsa (*langsa*)	0.827
Lhoksukon (*lhoksukon*)	0.826
mobilization (*mobilisasi*)	0.822
Medan (*medan*)	0.819
Bangladesh (*Bangladesh*)	0.813
resource person (*narasumber*)	0.813
Takengon (*tkn*)	0.811
let's go (*yok*)	0.811
Bulen (*bulen*)	0.807

These words can be understood as affecting the contexts in which *gempa* is discussed, and the similarity scores can be interpreted as the significance of the context in relation to each word. The computer model is thus able to show all the words in the corpus that are similar to each other. For the purpose of our analysis and based on the size of the data, we selected all the words which had a similarity score of 0.77 or higher. We arrived at this score after inspecting 50 most similar words for approximately 20 major keywords, and confirmed a similarity score of 0.77 as including all words that were deemed relevant to the analysis.^2^ Researchers who conduct qualitative research do this *intuitively*, which means they pick up far fewer words, but the AI‐based language model allows researchers to obtain a more systematic output of all related words based on the mathematical evidence of their high similarity scores. This makes it possible to turn over every stone to find the keywords in relation to other words. As a result, this form of research is more robust and more objective, and it produces more insights into the word under investigation than intuition‐based qualitative research.

### 2.1 Natural language processing and AI

This subsection explains how we created the AI‐based language model for researching the process of social capital formation and clarifies our argument as to why using AI is more effective than taking a traditional statistical approach. It begins by comparing classical text processing with more recent tools, including the AI‐based language model.

Before 2010, the use of computers in analysing texts was based on statistical models, which is called ‘natural language processing’ (NLP). From 2010 onwards, however, sophisticated computer algorithms known as ‘neural networks’ were being developed for the purpose of analysing texts—or even producing them. In 2022, for example, an advanced processing tool called GPT‐3 attracted a great deal of attention when it wrote a complete article that was published in *The Guardian* newspaper in the United Kingdom (GPT‐3, 2020). It is worth mentioning how much ChatGPT and other AI‐based language models have begun to impact our lives since then.

Statistical NLP was usually limited to identifying word co‐occurrences, or to deconstructing text into its grammatical components (known as part‐of‐speech analysis). While these models are computationally effective and useful for analyses of word co‐occurrences and grammatical components, they are unable to retain the contextual information for each word. That is, they lack model fidelity. When it comes to large bodies of text, AI‐based language models are able to keep track of every instance of how a particular word is used in every text, or how each word is ‘embedded’, rather than deconstructing text into its grammatical components.

Furthermore, our language model is trained *specifically* on the ‘local knowledge’ embedded in the interview transcripts and fieldnotes under investigation, and therefore is not affected by pre‐existing, perhaps dominating, and often Western‐centric general knowledge. This can be compared to language models such as GPT‐3 and GPT‐4, which are trained *generally* on the ‘general knowledge’ embedded in a large volume of general texts in English. The latter are frequently generated by those who can publish in English, which suggests that the ‘general knowledge’ may not be ‘general’ at all. In fact, ‘general knowledge’ may have disproportionately reflected the knowledge of the ‘haves’—in the context of this paper, donors of post‐disaster recovery assistance.

### 2.2 Word embeddings

Since 2014, the idea of neural networks has been applied to the study of ‘word embeddings’. Word embeddings are described as ‘one of the most popular representation[s] of document vocabulary’ because of their ability to capture so many aspects of vocabulary use, such as the ‘context of a word in a document, semantic and syntactic similarity, [and] relation with other words’ (Karani, 2018).

Word embeddings facilitate the analysis of structured and unstructured textual data by converting words into numerical representations. Rather than giving each word a single numerical value, words are represented as multidimensional vectors. In other words, they are transformed into a list of numbers, often incorporating several hundred at a time. This method of word‐to‐vector transformation makes it possible to capture the full extent of how the word is embedded. Words are then also compared with each other by looking at their neighbouring context words. This is done by ‘training’ a neural network. Once the training is complete, each word is assigned a unique vector representing its location relative to other words in the vector space. This results in the full *language model*, which contains all the vectors trained in relation to a specific body of text.

This method of training language models means that words can then be compared numerically. Words that occur more frequently within the same textual context are ‘closer’ to each other in the model, which can be expressed by their similarity score. This idea dates back to the distributional hypothesis first put forward by Harris (1954) in the 1950s, but it has only recently been turned into an actionable algorithm. Word similarities are computed by cosine similarity (that is, the similarity score), which measures the angle between vectors in a multidimensional vector space.

To illustrate how this works, we can imagine that most pre‐trained models could reliably perform tasks of the following nature:

Input: Tokyo – Japan + France

Output: Paris

By applying these (mathematically somewhat simple) measures, the cosine similarity enables us to perform vector arithmetic (Gittens, Achlioptas, and Mahoney, 2017). Word embeddings can thus be added to each other (context‐positive) or subtracted from each other (context‐negative), which shifts the predicted context or target word numerically within the vector space.

## 3 APPLYING THE MIXED‐METHODS APPROACH TO THE INDONESIAN CASE STUDIES

### 3.1 The dataset

In our research, we trained texts deriving from a total of 42 data files (interview transcripts and fieldnotes from participant observation). These were collected by MRN between April and October 2015, following two disasters in the Indonesian provinces of East Java and Aceh. Semi‐structured interviews and participant observation were conducted in each location. These data files consist of 25 interview transcripts (one transcript per interviewee) and 17 fieldnotes. MRN selected interviewees based on their diverse roles in post‐disaster recovery assistance: government officials, the FBO's representatives, village leaders, and community members. Fieldnotes are the researcher's reflections on and summary of participant observation and unrecorded interviews with study participants. The fieldnotes were taken only when MRN participated in communal activities such as harvesting, which means that there is no interview record as such. Furthermore, MRN could not necessarily interview all of the key informants; for instance, when one was too shy to be interviewed, or when an observed group dynamic in a community was important but not verbally mentioned by interviewees. This type of non‐verbal information is important in social capital analysis; interviews alone could not capture the features of social capital accurately. MRN's qualitative research is based on the same interview transcripts and fieldnotes. So, the dataset is the same across the qualitative and quantitative research, to allow for the revalidation of the findings from the qualitative research. The dataset remains in Bahasa Indonesia in both qualitative and quantitative studies.

In relation to the two case studies, the dataset consists of: 16 files relating to the Mount Kelud volcanic eruptions in East Java in February 2014; 22 files relating to the earthquake in the Gayo Highlands in Aceh in July 2013; and 2 files relating to both case studies (interviews with the FBO representatives discussing both the negative results at Mount Kelud and positive results in the Gayo Highlands and at another project site where the same FBO operates). The average word count of the files was 2,133.31 (σ = 2,782.66).

Questionnaires used in MRN's qualitative method were prepared at the outset of this research, but those for the purpose of the language model method were not, for two reasons. First, creating a questionnaire to suit the language model requires aligning the questionnaire with some keywords that were better trained in the language model, but doing so may jeopardise the quality of the questionnaire designed to explore local perceptions as its primary purpose. The second reason is to assess the applicability of this method for broader use. When the qualitative research is complete but researchers still need to validate or strengthen the result, they can utilise the AI‐based language models.

### 3.2 Ethnographic qualitative research: the gist of the argument

The case studies examine two disaster‐affected communities in Indonesia, in which an organisation called Dompet Dhuafa (Wallet for the Poor) worked to assist villages in the communities with relief and recovery efforts. Dompet Dhuafa is one of the largest humanitarian Islamic FBOs in Indonesia. MRN carried out a qualitative content analysis of the 42 data files using NVivo, a computer programme that enables researchers to undertake qualitative content analysis by coding textual data. Using the software, he developed several coding classifications for the analysis to represent specific attributes of the case study sites (people, places, and local customs), the types of social capital created (bonding, bridging, and linking), the process of social capital formation (smooth and contested), and the agencies involved (the government, the FBO, and disaster‐affected communities). He then used these classifications as codes to analyse each of the texts and arrive at his results. He also did additional research in 2021 and 2023 to update his data and confirm his findings.

MRN's principal finding is the importance of two key factors in post‐disaster aid delivery, both of which directly affect the ways in which social capital is strengthened, or not: the function of the village facilitator; and the existence of religiously‐based communal activities. These two factors were instrumental in the smooth (positive) process of social capital formation that occurred in the aftermath of the earthquake in Aceh. Here, the village facilitator played a significant role in creating a ‘linking’ social capital by establishing a good relationship between Dompet Dhuafa as aid provider and the villagers as aid recipients. The village facilitator also strengthened the ‘bonding’ social capital between the villagers by utilising religiously‐based communal activities and formulated a ‘bridging’ social capital by expanding community networks.

The other case study community experienced the volcanic eruption of Mount Kelud, but the social capital formation process was contested. This was largely because of the absence of an efficient village facilitator, and the FBO's lack of understanding of the importance of religiously‐based communal activities. These factors arguably led to contestation between the village leader and the FBO's facilitator over the legitimacy and efficacy of the organisation's activity in that village. The process of creating a ‘linking’ social capital as performed by the village facilitator in the disaster‐affected village led to complaints by the village leader about the former's lack of communication and appreciation of local norms in relation to providing updates on project activities to the village leadership. Although a ‘bonding’ social capital was strengthened within the community, there was an issue with community participation, as the aid was initially delivered to some selected community members only. Lastly, the village facilitator failed to generate any ‘bridging’ social capital owing to his absence from the village. As a result, the relationship between Dompet Dhuafa and the villagers came to an end.

### 3.3 An AI‐based language model: the research process

Against this background of the results produced by MRN's qualitative analysis, our main aims in applying the AI‐based language model were (i) to validate the results by using a different methodology and (ii) to examine whether the model could offer any additional insights for researchers. Keeping these objectives firmly in mind, we then set about creating the AI‐based language model that we needed for our analysis.

**FIGURE 1:**
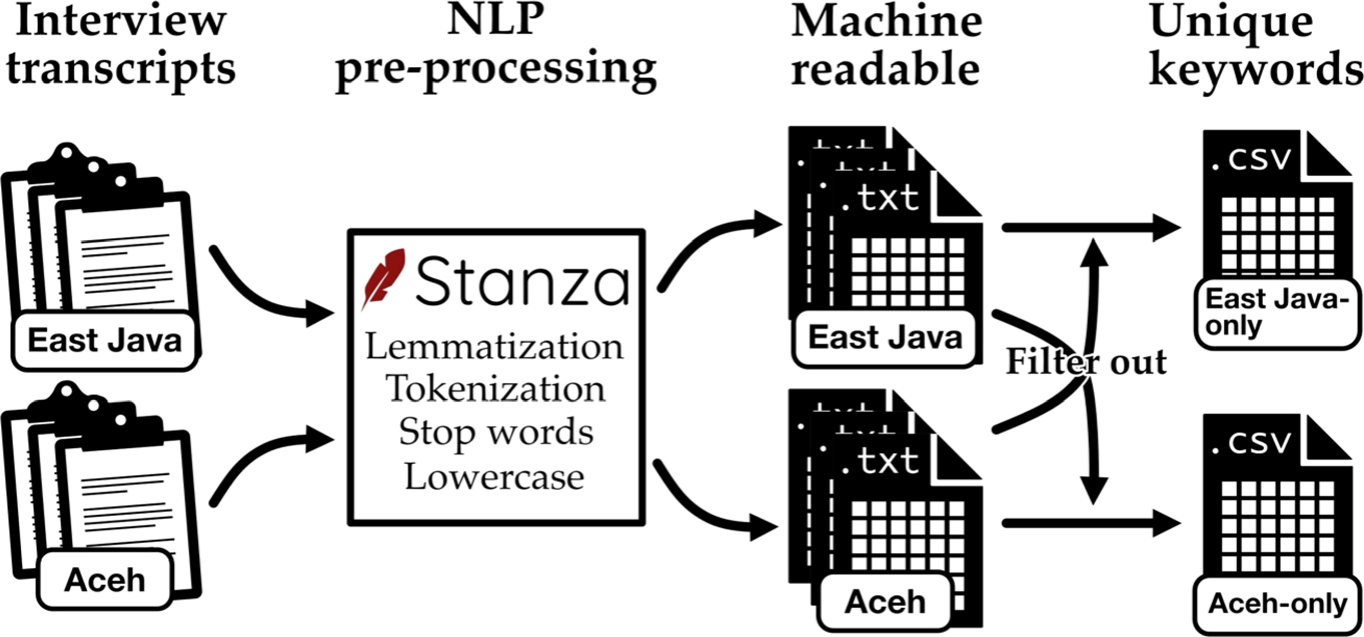
Process to transform interview transcripts and fieldnotes into a set of individual keywords. **Source:** Daniel Moritz Marutschke.

Figure 1 shows the process of transforming interview transcripts and fieldnotes into a set of individual keywords. The data (interview transcripts and fieldnotes) were converted into plain text files and manually tagged for machine processing (East Java or Aceh). Afterwards, the data were ‘NLP pre‐processed’, which means that they were aggregated and split into individual words through NLP—that is, part‐of‐speech and morphological analysis—for which we used a multilingual NLP tool called Stanza. In this process words were converted into their base form (called ‘lemmatization’ and ‘tokenization’). To prevent the computer from analysing the usual linguistic links between nouns and articles or verbs and prepositions, called ‘stop words’, these were then removed using the Natural Language Toolkit, which provides lists of such ‘filler’ words in several languages, including Bahasa Indonesia (Bird, Klein, and Loper, 2009). Other fragments, such as punctuation and special characters, were removed manually, and misspelled words were corrected. The resulting (pre‐processed and filtered) textual data were then saved as individual plain text files. All texts were converted into lower case. These rendered the data as machine‐readable text files. Two CSV (comma‐separated values) files were created, which contained words only present in texts tagged ‘East Java‐only’ or ‘Aceh‐only’, respectively.

**FIGURE 2:**
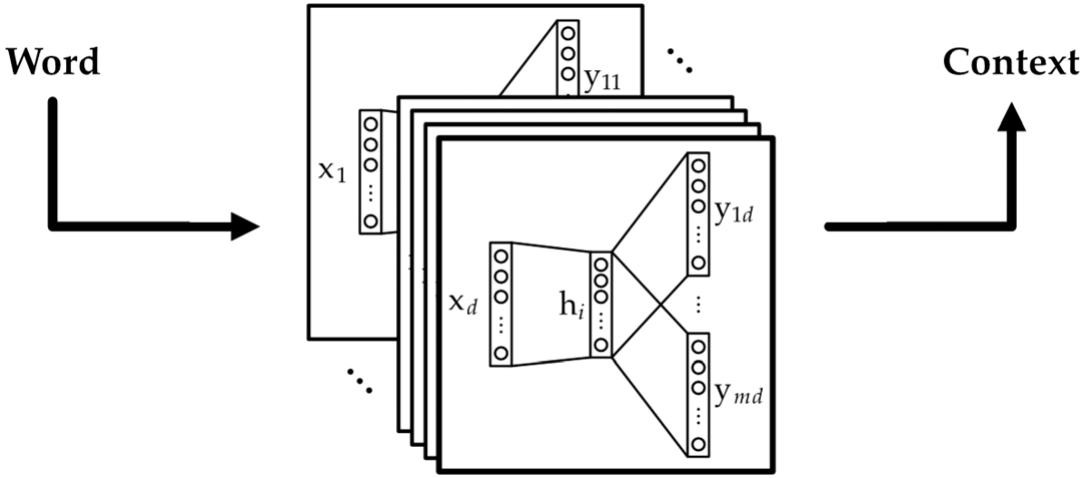
Conceptual visualisation of how Word2Vec neural networks transform word to context. **Source:** Daniel Moritz Marutschke.

We then trained the AI‐based language model to recognise the word embeddings in our database using the Gensim model implementation of Word2Vec in Python (Rehurek and Sojka, 2011), seeking words connected with a particular context: the social capital formation process as ‘contested’ or ‘smooth’ (see Figure 2). This model yielded a vocabulary of 5,698 unique words. Word embeddings are trained by using Word2Vec with *x*_*d*_ as an input word and *y*_*md*_ as an output word sequence. The number of unique words in a dataset is denoted by *d*. The output has *m* number of words, double the window size *t*. The window size equals the number of words that Word2Vec is ‘looking’ at to the left and right of each word in the text for context information. A hidden layer in the neural network retains this context information throughout the training process and returns a vector of size *i*, the vector size of a word.^3^ We corrected typos and synthesised multiple spellings manually after looking at the model results (for example, ‘jogya’ can be spelt ‘jogja’ depending on the region). A short code was then included to correct automatically the typographical errors or to replace regional variations in spelling with a standard rendering for the next phase of analysis.^4^

**FIGURE 3:**
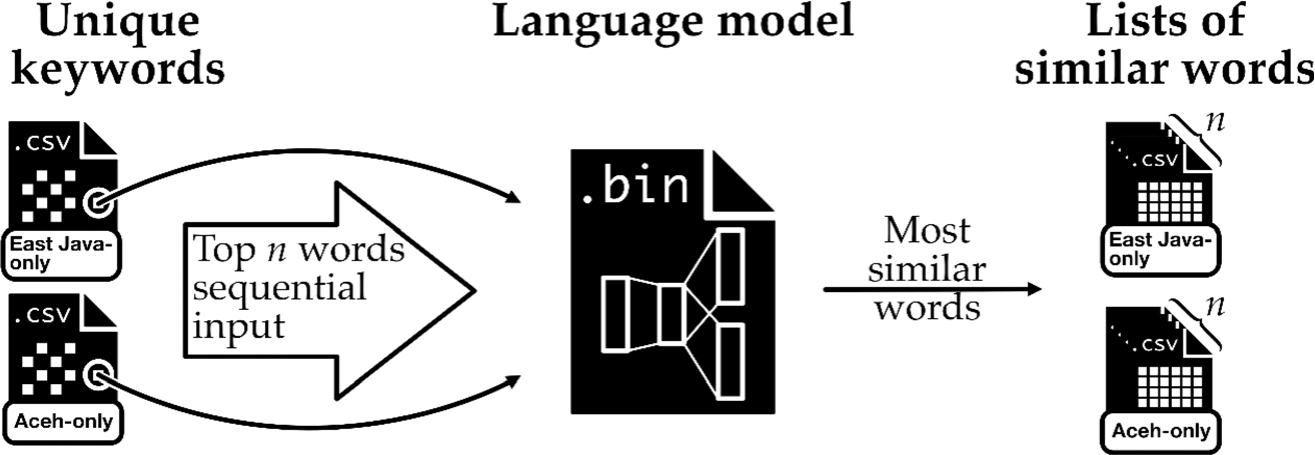
Process to create lists of similar words from unique keywords. **Source:** Daniel Moritz Marutschke.

As we began to identify words from the interview transcripts and fieldnotes that only occurred in the context of either contested or smooth social capital formation, these words were filtered out and stored in a machine‐readable spreadsheet format (CSV) as ‘unique keywords’ (see Figure 3). This is because words that appear in only one category may provide more precise information about the nature of that category than words that appear in both categories. As Figure 3 demonstrates, each unique keyword (top *n* words) on the spreadsheet was processed sequentially through the AI‐based language model, which produced lists of all similar words (with their similarity scores) for each input. MRN then used these lists to validate his qualitative analysis. The lists also made it possible to explore some previously undetected elements, as discussed below.

Using this process, our AI‐based language model produced lists of more than 100 similar words in the two contexts (‘contested’ and ‘smooth’) in the East Java and Aceh case studies, respectively. From these lists, words that were *most relevant* to the contested and smooth process of social capital formation—in other words, keywords—were then manually selected. Tables 1 and 2 are condensed tables that present keywords with the highest similarity scores (0.77 or higher) that were used to describe the contested or smooth nature of social capital formation, respectively.

**TABLE 1:** Similarity table for the East Java case study.

Keyword	Meaning	Frequency	Language model: similar words and similarity scores	Types of social capital
**Scene**				
klangon	The name of a hamlet in Pandansari village where the disaster recovery programme took place. Community polarisation happened here (name of hamlet)	150	cemburu / jealous (0.930)nerima / accept (0.918) dusun / hamlet (0.914)protes / protest (0.904) ketut / ketut (another hamlet in Pandansari village) (0.899) survey / survey (0.894) datanga / coming (0.887)	N/A
pandansari	The name of the village that was the site of the disaster recovery intervention in East Java	76	wisata / tourism (0.912)desa / village (0.889) huni / inhabit (0.875)tetangga / neighbour (0.868) sari / sari (0.862) aiyub / aiyub (one of the individuals outside the village asked to help) (0.851) pj / pj (abbreviation from penjabat, acting) (0.846)	N/A
**Elements to maintain or formulate social capital**				
saiful	The Dompet Dhuafa representative from Jakarta who managed the recovery assistance programme to Klangon	26 [stet]	oke / okay (0.922) oo / and (0.893) sampek / until (0.883) baro / from barokah, meaning blessing (0.858) ee / yes (0.848) em / em (mumbling) (0.842) mantau / monitoring (0.839)	Linking social capital
senam	Aerobic health activity initiated by the Pandansari village leader; a form of social capital	49	manteb / good (0.963) 27 / 27 (refers to the date when senam took place) (0.937) nyaman / comfortable (0.935) lomba / contest (0.932) diam / silent (0.924)sebetul / really (0.922) sarah / sarah (name of Pandansari village head) (0.918)	Bonding social capital
pkk	Women's organisation set up by the local leadership; a form of social capital	39	lpmd / lpmd (abbreviation from Lembaga Pemberdayaan Masyarakat Desa, village community empowerment body) (0.972) bpd / bpd (abbreviation from Badan Permusyawaratan Desa, village consultative body) (0.950) darimana / from where (0.908) perbub / change (0.907) sarah (name of Pandansari village head) (0.893) pinter / smart (0.892) sakirman / sakiman (name of the husband of the village head) (0.891)	Bonding social capital
hentraktor	Hand tractor; the assistance provided by Dompet Dhuafa; a form of social capital	27	lambat / slow (0.933) aktifitas / activity (0.930) sayur / vegetables (0.930) emm / emm (mumbling) (0.928) bantuan / grant (0.925) ntuk / meaning untuk, for (0.919) solusi / solution (0.918)	Linking social capital
barokah	The name of the farmers' group (livestock); the result of social capital formation	12	saiful:oo / saiful: Oh (0.993) searching / searching (0.993) rintis / initiate (0.993) bawahi / underline (0.992) sedih / sad (0.992) saiful: / saiful (name of Dompet Dhuafa representative): (0.992) saiful:ya / Saiful: yes (0.992)	Bonding social capital
traktor	Tractor; the assistance provided by Dompet Dhuafa; a form of social capital	12	alisasi / no more (0.971) sehari–hari / daily (0.970) nol / nol (0.969) prasarana / infrastructure (0.968) other2 / other2 (0.964) peternak / breeder (0.963) singkat / short (0.962)	Linking social capital
**Elements to damage social capital**				
sarah	The name of the village leader of Pandansari in East Java, who was in contestation with Muhammad, the Dompet Dhuafa village facilitator who was supposed to stay in the village	534	bu / Madam (0.939) unk ya / unique (0.905) unk unk / unk unk (0.903) senam / gymnastics (0.879) darimana / from where (0.859) ngutang / borrow (0.858) 27 / 27 (0.854)	N/A (similar words are not indicative)
muhammad	The name of the Dompet Dhuafa village facilitator who was supposed to stay in the village but did not and was not welcome by the village leader, Sarah	446	kasun / head of hamlet (0.887) suara / voice (0.882) dehem / clear throat (0.881) batuk / cough (0.881) aa / yes (0.876) tertawa / laugh (0.873) unk ya / unique (0.860)	N/A (similar words are not indicative)
lurah	The Indonesian term for the head of the village, who was in contestation with the village facilitator	33	sampean / you (0.949) remaja / teenagers (0.936) nakal / mischievous (0.929) pesta / party (0.925) nyambangi / visiting (0.921) selamat / safe (0.921) diam / silent (0.921)	N/A (similar words are not indicative)
protes	The negative consequence of aid delivery, which resulted in protest; negative social capital	23	ketut / ketut (another hamlet in Pandansari) (0.984) sekelompok / a group (0.957) cemburu / jealous (0.954) survey / survey (0.946) nerima / accept (0.945) kedusun / head of hamlet (0.943) rinci / detail (0.940)	Bonding and linking social capital
kasun	The Indonesian term and acronym for head of hamlet (kepala dusun). The contestation between the former and current kasun resulted in polarisation; negative social capital	21	aa / yes (0.963) lambat / slow (0.962) ngurus / manage (0.960) kokok / crow (0.955) gulir / revolving (0.953) ntuk / for (0.952) dehem / clear throat (0.950)	N/A (similar words are not indicative)
tegur	To give a warning: by the head of village to Muhammad, the village facilitator; negative social capital (similar to protes, tegur is stronger and more directed)	15	giru / that's it (0.970) munjung / munjung (another hamlet in Pandansari) (0.964) dengar / listen (0.964) indra / sense (0.962) niat / intention (0.961) sungkan / reluctant (0.961) blumpat / blumpat, meaning Plumbang (another hamlet in Pandansari) (0.959)	Linking social capital

**Notes:** the seven most similar words from each keyword are listed with their similarity scores rounded to three decimal points. N/A = not applicable.

**Source:** authors.

**TABLE 2:** Similarity table for the Aceh case study.

Keyword	Meaning	Frequency	Language model: similar words and similarity scores	Types of social capital
**Scene**				
jaluk	The name of the village that is the aid recipient	193	timur / East (0.843) ketol / Ketol (name of subdistrict) (0.822) datu / Datu (refers to Datu Jaluk, the villagers' ancestor) (0.817) nopen / Nopen (0.811) mesjid / mosque (0.803) induk / parent (0.791) masjid / masjid (0.784)	N/A
gempa	Earthquake	179	2013 / 2013 (the year when the studied earthquake happened) (0.876) meriah / meriah (a word match for Bener Meriah, the name of the district that was also affected by the 2013 earthquake) (0.861) jogya / jogya (Yogyakarta, another locality in Java affected by the earthquake in 2006) (0.838) sejarah / history (0.837) konflik / conflict (0.832) henra / Hendra (one of the Dompet Dhuafa representatives who came first to Jaluk as part of emergency response following the earthquake) (0.828) bantul (a part of Yogyakarta where the earthquake happened) (0.827)	N/A
**Elements to maintain or formulate social capital**				
Jodi	The name of the Dompet Dhuafa representative from Jakarta who helped with social capital formation	377	zhock / zhock (refers to ZOPP, the planning method learned by Dompet Dhuafa staff) (0.886) satria / Satria (name of the Dompet Dhuafa representative) (0.865) ipb / IPB (Bogor Agriculture Institute) (0.841) bogor / Bogor of West Java (the location of one of Dompet Dhuafa's offices) (0.831) jerman / Germany (refers to where the ZOPP method comes from) (0.829) tukang / craftsman (0.821) struk / invoice (0.819)	Linking social capital
mulyadi	The name of the youth leader who welcomed Nopen, the village facilitator	131	wawancara / Interview (0.854) fatah / fatah (name of Jaluk villager) (0.845) rujuk / consult (0.841) bg / bg (shortened from bang, which means brother) (0.826) tembak / shoot (0.824) keuchik / keuchik (village head) (0.823) pemuda / youth (0.810)	Linking and bonding social capital
muslim	Indication of using religious identity as a part of social capital formation. Aid from Dompet Dhuafas to Muslim recipients	87	non / non (0.939) manusia / human (0.915) agama / religion (0.904) infak / infaq (Islamic donation) (0.900) gereja / church (0.893) shadaqah / shadaqah (Islamic donation) (0.886) himpun / collect (0.885)	Linking and bonding social capital
gotongroyong	Communal work in the village; a form of social capital	66	jumat / Friday (0.931) adak / organise (0.930) hahaha / hahaha (laugh during interview) (0.925) haha / haha (laugh during interview) (0.889) setor / sector (0.888) mengkoordinir / coordinate (0.883) ajak / Invite (0.882)	Bonding social capital
nopen	The name of the Dompet Dhuafa facilitator who stayed in the village and was welcomed by villagers, mainly Mulyadi	49	adik / younger brother or sister (0.909) yudhi / yudhi (name of Jaluk villager) (0.908) cek / uncle (0.904) zona / zone (0.900) putra / putra (name of Jaluk villager) (0.895) sekretaris / secretary (0.892) definitive / definitive (0.890)	Bonding social capital
gapoktan	Farmers' association; the result of social capital formation	36	rasyid / rasyid (name of Jaluk villager) (0.918) paksaan / compulsion (0.895) eemn / eemm (mumbling) (0.871) kata–kata / words (0.869) catat / note (0.860) masing–masing / each (0.857) terserah / up to you (0.857)	Bonding social capital
puasa	Fasting, or the fasting month of Ramadan, which can serve as a form of social capital	36	lebar / wide (0.930) capek / tired (0.915) cuti / holiday (0.906) besok / tomorrow (0.904) tab / tab (a sound made during drying of the coffee bean) (0.904) marathon / marathon (0.889) kakek / grandpa (0.883)	Bonding social capital
shalat	Prayer; a religious activity which can serve as a medium for social capital formation; where people are gathering to perform the activity	36	jamaah / adherents (0.908) medan / Medan (capital city of North Sumatera) (0.907) tarawih / tarawih (night prayers during Ramadan fasting month) (0.903) hendra / Hendra (one of the Dompet Dhuafa representatives who came first to Jaluk as part of emergency response following the earthquake) (0.893) mahasiswa / university student (0.893) rekomendasi / recommendation (0.892) rujuk / consult (0.892)	Linking social capital
kebersamaan	Togetherness; positive social capital	33	105 / 105 (meaning IDR 105,000, which refers to the price of fertiliser) (0.963) kompak / unified (0.958) dikali / multiplied (0.956) skusi / meaning diskusi (to discuss) (0.955) ibu–ibu / mothers (0.951) hehehe / hehehe (laugh during interview) (0.945) diakhir / at the end (0.944)	Bonding social capital
meugang	Cultural activity before and on the completion of the fasting month; a form of social capital (only in Aceh)	21	kerbau / buffalo (0.958) daging / meat (0.950) nenek / grandmother (0.946) kilo / kilogram (0.943) 400 / 400 (meaning IDR 400,000 cash assistance received) (0.941) mupuk / fertilise (0.941) kg / kg (0.939)	Bonding social capital
adat	Customs; a form of social capital (universal custom/tradition)	18	wvi / wvi (World Vision Indonesia) (0.980) mdmc / mdmc (Muhammadiyah Disaster Management Center) (0.975) lintas / cross (0.975) legalitas / legality (0.968) ytbi / ytbi (Yayasan Tanggul Bencana Indonesia, one of the Indonesian faith‐based organisations) (0.968) bumi / earth (0.967) yakkum / yakkum (Yayasan Kristen untuk Kesehatan Umum, one of the Indonesian faith‐based organisations) (0.966)	Linking social capital
kompak	Togetherness; positive social capital	18	1,000 / 1,000 (0.975) sepenuhnya / completely (0.972) usia / age (0.966) presentase / percentage (0.964) selesai / done (0.962) kata–kata / words (0.962) dikali / multiplied (0.961)	Bonding social capital
musarapakat	The name of the gapokton/group (farmers' association); the result of social capital formation	18	tanggungjawab / responsibility (0.917) ituk / itu (it) (0.917) saham / share (0.916) gairah / excitement (0.910) duafa / duafa (from Dompet Dhuafa) (0.906) senilai / worth (0.904) gm / gm (general manager) (0.904)	Bonding social capital
musholla	A general prayer hall; a place for social capital formation	3	polindes / polindes (abbreviation from pondok bersalin desa, village delivery house) (0.993) kimpun / the bouquet (0.986) dsp / dsp (abbreviation from dana siap pakai, disaster emergency budget) (0.985) setau / know (0.984) tandatangi / sign (0.984) rawih / rawih (from tarawih, night prayers during Ramadan fasting month) (0.984) mck / mck (abbreviation from mandi, cuci, kakus, bathing, washing, toilet) (0.984)	Bonding social capital
masjid	Mosque; a venue for social capital formation (universal name for mosque)	1	mesjid / mosque (0.914) tarawih / tarawih (night prayers during Ramadan fasting month) (0.881) kayu / wood (0.866) ibu2 / mothers (0.866) pokmas / community groups (0.864) datu / datu (name of Jaluk village ancestor) (0.863) dayah / Islamic boarding school (0.863)	Bonding social capital
**Elements to damage social capital**				
konflik	Aceh as post‐conflict area; indicates low stock of social capital (pre‐existing condition)	62	bangladesh / Bangladesh (0.920) meriah / meriah (a word match for Bener Meriah, the name of the district that was also affected by the 2013 earthquake) (0.907) steek / stakeholder (0.902) adat / adat (local customary law) (0.901) bantul (a part of Yogya where the earthquake happened) (0.899) sejarah / history (0.897) jogya / jogya (Yogyakarta, another locality in Java affected by the earthquake in 2006) (0.896)	Bonding social capital

**Notes:** the seven most similar words from each keyword are listed with their similarity scores rounded to three decimal points. N/A = not applicable.

**Source:** authors.

In brief, this research trains a language model on the data by using the ‘word embedding’ training process, while applying quantitative and qualitative processes iteratively, as follows:

1. The data are trained based on all interview transcripts and fieldnotes (quantitative process).
2. The result of the trained data is checked manually to identify unique keywords—unique to a specific context. The only word that defines the keyword or a word that relates to the contested or smooth nature of social capital are selected (qualitative process).
3. The unique keywords then create lists of similar words (quantitative process).
4. The result (lists of similar words) is manually analysed (qualitative process).
5. An in‐depth evaluation with MRN's research processes and results is performed. Two independent researchers (a computer science specialist and a scholar working on disaster responses, both of whom have no direct history of fieldnotes) critically re‐evaluated MRN's interpretations, thereby attempting to reduce bias as much as possible qualitatively.

The above process means that the mixed approach that this paper explains is not just a simple combination of qualitative and quantitative methods. It is an *iterative process* of initial qualitative ethnological research and quantitative research based on a language model.

The following section analyses the tables consisting of unique keywords, the frequency of the unique keywords, similar words, similarity scores, and types of social capital that the similar words indicate. By using these tables, we attempt to answer the three research questions posed in our introduction in two steps. The first step focuses on the ‘means’ by which social capital is formulated or damaged, by analysing ‘keywords’, ‘meaning’, and ‘frequency’ (the left three columns of Tables 1 and 2). The second step focuses on the ‘ends’ for which social capital is formulated or damaged—in other words, which type of social capital was formulated or damaged in relation to each of the keywords—by analysing the language model output (the penultimate column of Tables 1 and 2).

## 4 RESULTS

### 4.1 Research results from an AI‐based language model: keywords and frequency

The unique keywords in Bahasa Indonesia, their meaning in English, and their frequency in Tables 1 and 2 alone help us to validate the results of the qualitative research. The keywords can be interpreted as the ‘means’ to formulate or damage social capital. The frequency shows the number of times the keywords are mentioned across all data. More mentions of a particular keyword may reveal the importance of the keyword in the perception of the interviewees, or this may simply be a reflection of the length of an interview in which the keyword was cited. Researchers need to go back to the interview data to ascertain the probable explanation. A comparison of Tables 1 and 2 shows what distinguished the two communities in terms of the process of social capital generation—whether it was smooth or contested. In contrasting them, this subsection aims to validate, and if possible, add extra insight to, the findings of MRN's qualitative research. It begins with a discussion of frequent keywords in each table to demonstrate the more important ‘means’ of social capital formation from the perspective of local community members. It then compares the two tables to discern commonality and difference.

Table 1 is a similarity table in the East Java case study.^5^ Pandansari village in East Java has seven hamlets, one of which is Klangon, where Dompet Dhuafa undertook its post‐disaster recovery aid programme. Table 1 shows two keywords about the scene (klangon and pandansari), six about ‘elements to maintain or formulate social capital’, and another six about ‘elements to damage social capital’.

The keywords, and their meaning and frequency in Table 1 suggest two elements that contributed to the contested process of social capital formation in this case study: key personnel and communal activities.

The three most frequent keywords are the following key individuals who were related to Dompet Dhuafa's post‐disaster recovery assistance:

- Sarah, the village leader of Pandansari, who was in contestation with Muhammad, Dompet Dhuafa's facilitator (534 mentions);
- Muhammad, a Dompet Dhuafa facilitator who was supposed to stay in the village, but chose not to do so (446 mentions); and
- Saiful, the representative of Dompet Dhuafa from Jakarta, who managed the project to deliver recovery assistance to Klangon (266 mentions).

In comparing the counts of keywords, Sarah and Muhammad, being ‘elements to damage social capital’ (see Table 1), received an overwhelming number of mentions, far more than those of Saiful, being an ‘element to maintain or formulate social capital’ (see Table 1). This reveals clearly that these two individuals and their contestation led to social capital formation in a negative manner. We then went back to the files to see whether the interviews in which Sarah and Muhammad were mentioned were significantly longer than those that mentioned Saiful. However, these three interviewees were mentioned by the same interviewees most times, so the number of mentions represents the significance of these individuals to the interviewees' perceptions. This finding is congruent with the research result presented in the abovementioned gist of MRN's paper, thereby validating that research result.

The second element that contributed to the process of social capital formation in East Java, which was not negative in itself, was the use of local activities and local organisations:

- Local activities and donation of goods, both of which enabled villagers to interact—for example, aerobic health activity (*senam*) and a donation of farm tractors (*hentraktor and tractor*), which a farmers' group called Barokah received from Dompet Dhuafa (49 and 39 mentions, respectively).
- Local organisations such as the Pemberdayaan Kesejahteraan Keluarga (PKK) (Family Welfare Empowerment, a women's organisation set up by local leadership) and Barokah (or formerly Kelud Barokah, a livestock farmers' group) (39 and 12 mentions, respectively).

These activities brought community members together, which maintained social capital in the Klangon hamlet.

As for the Aceh case study, a post‐disaster recovery programme took place in Jaluk village in the aftermath of an earthquake. Table 2 shows that the overwhelming majority of keywords relates to ‘elements to maintain or formulate social capital’. The only keyword that pertains to ‘elements to damage social capital’ is ‘conflict’ (*konflik*), as this case study was conducted in post‐conflict Aceh.

As in the East Java case study, Table 2 suggests that the most frequently mentioned keywords are the key individuals related to Dompet Dhuafa's post‐disaster recovery assistance. However, the nature of the following individuals in Aceh is quite opposite to that of Sarah and Muhammed in East Java:

- Jodi, the representative of Dompet Dhuafa from Jakarta (377 mentions).
- Mulyadi, the youth leader who welcomed Nopen, a Dompet Dhuafa facilitator (131 mentions).
- Nopen, a Dompet Dhuafa facilitator (49 mentions).

The number of mentions each of Jodi, Mulyadi, and Nopen shows that the effective FBO representative from Jakarta (Jodi) and the local community leader (Mulyadi) are among the top two most significant variables in the perceptions of community members; the village facilitator (Nopen) is the fifth most significant. All of these individuals worked to contribute to the smooth formulation of social capital, but the roles that Jodi played were mentioned far more frequently than those of the other two. This is because Mulyadi and Nopen, in their respective interviews with MRN, cited Jodi as the central figure who contributed to formulating social capital in the disaster‐affected village.

The second element that further supports the process of social capital formulation is religion, communal activities, norms, and community organisations:

- Muslim (87 mentions).
- Religiously‐based communal activities that created an environment in which local villagers could interact, such as communal work in the village (*gotongroyong*) (66 mentions), fasting (*puasa*) (36 mentions), prayers (*shalat*) (36 mentions), fasting (*puasa*) (36 mentions), shalat (*prayer*) (36 mentions), and cultural activity before and after the Ramadan fasting month (*meugang*) (21 mentions).
- Some normative terms showing togetherness (*kebersamaan* and *kompak*)^6^ (33 and 18 mentions, respectively).
- Farmers' association (*Gapoktan*) and *Musarapakat* (the name of the association) (36 and 18 mentions, respectively).

These show that the means to formulate social capital went beyond local organisations and local activities to include *religiously‐based* communal activities that bring people ‘together’. These, again, validate the findings of MRN's qualitative research.

In examining the category of ‘elements to maintain or formulate social capital’ in Tables 1 and 2, what is common to both is the importance of the FBO representative: Jodi in Aceh and Saiful in East Java were by far the most frequently mentioned keywords in that category (377 and 266 times, respectively). This also provides additional insight into MRN's qualitative analysis. The qualitative research argues that the existence of effective village facilitators matters to the smooth formation of social capital, but in both case studies, the representative of the FBO is the most frequently mentioned, irrespective of whether overall social capital formation is contested or smooth. In other words, how the representatives are perceived in the community also matters to the formation of social capital—this, too, is an additional insight that the language model offers to MRN's qualitative research. This attests to the importance of leadership in bringing people together, which is emphasised in the literature on social capital formation (Roberts, 2013).

Two clear differences between the two case studies are: whether the relationship between a village leader and a local facilitator was smooth or contested; and the extent to which communal activities were linked to the religion of the community. Whether these two are both necessary to formulate social capital smoothly, or whether one or other is sufficient requires more analysis, but these two factors relate to the distinctly different natures of the process of social capital formation in the two communities.

### 4.2 Research results of an AI‐based language model: similarity scores

When examining the lists of word similarity (the penultimate column of Tables 1 and 2), MRN checked every word that the language model picked up as ‘similar’. Each keyword returned hundreds of ‘similar’ words. After selecting the first 50 words that have the highest similarity scores, MRN went through the 50 words and selected the words that fit the following criteria:

1. A word that defines the original word (for instance, an original word, *Muhammad*, in Table 1 is the name of the FBO's facilitator, who was head of a hamlet. The word with the highest similarity to Muhammad is *kasun*, which means ‘head of hamlet’).
2. A word that relates to the nature of social capital (for example, an original word, *klangon*, in Table 1 is the name of the hamlet where a disaster recovery programme took place, but which led to community polarisation. The word with the highest similarity to *klangon* is *cemburu*, which means ‘jealous’).

The list of selected words is shown as ‘similar words and similarity scores’ in Tables 1 and 2. This helped us to validate the result of the earlier qualitative analysis. We did so by checking whether the qualitative analysis addressed every similar word *that the researcher considers relevant to the analysis*. Using the example of *klangon* (the name of the hamlet) in Table 1, similar words are, in order of similarity score, jealous, accept, hamlet, protest, *ketut*, survey, and coming. These similar terms can be categorised in three ways:

1. MRN considered jealous, hamlet, and protest to be relevant words to describe the *contested* process of social capital formation. He then re‐evaluated the importance of each word to ascertain whether the same words have been addressed in his qualitative work.
2. Other words, such as accept, *ketut*, survey, and coming, provided MRN with a chance to reconsider whether he could analyse the contested process of social capital formation from the perspective of those words:
  - if MRN still considers those words to be irrelevant to the case study, he stops considering them further.
  - if MRN discovers the relevance of those words, or is unsure about their relevance, he goes back to his own question to ensure that the word is not an artefact of the question asked. This makes the iterative process of mixed methods more robust. He then reconsiders the relevance of those words to add extra insights.

The iterative process revalidates the original qualitative research findings by confirming relevant themes, and adding extra insight by discovering other similar words that might not have been noticed without running the AI‐based language model. Furthermore, we analysed the overall tendency of the similar words of unique keywords in relation to the operational definition of social capital—bonding, bridging, and linking—to answer the research question about how the FBO formulated social capital in these two case studies.^7^ Similarity scores reflect numerical closeness between two embedded words. The more often a pair of words occurs together in the text data, the higher the similarity score of these two words generally. We also analysed whether similar words indicate any of the three types of social capital. For example, words similar to Jodi (the Dompet Dhuafa representative in Aceh (Table 2)) are *zhock* (a planning method), Satria (a name of Dompet Dhuafa representative), IPB (Bogor Agriculture Institute), Bogor of West Java (a place name), Germany, craftsman, and invoice. These words tend to show that Jodi is discussed in the context of linking various persons and organisations. This is indicative of ‘Jodi’ relating to ‘linking’ social capital. As another example, words similar to Nopen (the name of the Dompet Dhuafa facilitator) include brother/sister, uncle, and names of Jaluk villagers. This is indicative of ‘Nopen’ relating to ‘bonding’ social capital. What follows below explains these points in more detail empirically.

When examining the words similar to all of the keywords listed in the tables, we analysed the keywords that pertain to scene in the tables (Klangon and Pandansari in Table 1 and Jaluk and *gempa* in Table 2), and the two elements identified in the previous section: key personnel and communal activities.

In relation to scene, the similar words show the nature of the disaster‐affected community. In East Java (see Table 1), Klangon hamlet is associated with such words as jealous, accept, hamlet, protest, and Ketut hamlet (which is Klangon's neighbour that did not receive Dompet Dhuafa aid). These terms demonstrate the mixed nature of smooth and contested social capital formation within this hamlet, and correspond to the list of keywords in Table 1.

Apropos of the ‘elements to maintain or formulate social capital’, by means of Saiful's management of the project, some local activities, and local organisations, Klangon hamlet strengthened its social capital through bonding. By ‘monitoring’ (Saiful's similar word) the project, Saiful also strengthened social capital through ‘linking’ the hamlet with Dompet Dhuafa. However, similar words for ‘sarah’, ‘muhammad’, and ‘lurah’ (the Indonesian term for head of village) do not reveal anything particularly meaningful. This can be interpreted as evidence of social capital formation being unrecognisable.

In Aceh (see Table 2), Jaluk village is associated with Timur (the eastern part of Jaluk), Ketol (the name of the subdistrict in which Jaluk is located), Datu (which refers to Datu Jaluk, the ancestor of Jaluk village), Nopen (the Dompet Dhuafa facilitator), mesjid (mosque), parent, and masjid (also mosque; *masjid* and *mesjid* are used interchangeably). These terms demonstrate the significance of some communal activities based on religion, which, again, correspond with the list of keywords in Table 2. Nopen as the Dompet Dhuafa village facilitator played an important role; mosque was important; and Datu (the ancestor) refers to kinship—an important feature of the communal structure confirmed in this similarity list. In sum, the list of similar words in the scene confirms the findings of the analysis of keywords and their frequency, as well as the findings of MRN's qualitative research, which shows the overall nature of social capital formation in these two cases.

In terms of key individuals, a comparison of the similar words of Saiful, Sarah, and Muhammad in East Java (see Table 1) and those of Jodi, Mulyadi, and Nopen in Aceh (see Table 2) highlights a very interesting tendency. The key individuals listed in Table 1 are all associated with random words, and any various mumbling, coughing, and throat clearing. These words (sounds) themselves do not allow us to arrive at any finding. However, the similar words in Table 2 show some remarkable differences from those in Table 1. Jodi's similar words consist of many meaningful names, such as outside organisations (Bogor Agriculture Institute), a particular planning method, various villagers' names, and the use of words that demonstrate friendliness (brother, sister, and uncle). These reveal that the Aceh case shows that these three individuals contributed to social capital formation by ‘linking’ with outside organisations, Dompet Dhuafa's ‘linking’ with villagers, and ‘bonding’ between villagers by enhancing the degree of friendliness. They confirm one of the key arguments of MRN's qualitative research: effective village facilitators help to formulate social capital smoothly.

Furthermore, one of the additional insights was that whether the village facilitator was originally an insider or outsider did not matter so much. The similar words of these people in both case studies did not demonstrate any reference to outsider or insider. In the East Java case (see Table 1), for example, Saiful and Muhammad were outsiders and Sarah was an insider, but Saiful is listed as an element to maintain or formulate social capital, while Muhammad and Sarah are listed as elements that damage social capital.

As for communal activities, a comparison of the two tables reveals religious significance. We compared the elements listed in the keywords analysis. In other words, in Table 1, we analysed word similarity relating to local activity and the donation of goods, and local organisations. In Table 2, we analysed Muslim, religiously‐based communal activities, normative terms, and the farmers' association. A clear difference is evident in Muslim‐related activities in the Aceh case: for instance, similar terms include Friday, Ramadan prayer, buffalo and meat, *infaq* (one form of Islamic donation), prayer room, church, and *shadakah* (another form of Islamic donation, particularly on a voluntary basis), all showing that religiously‐based communal activities help to formulate bonding. The other genre is related to community building: for example, coordinate, necessitate, compact, participate, togetherness, grouped, responsibility, and join, all of which, again, point up some of the degree of importance in communal activity.

These words confirm the key argument of MRN's qualitative research: the key role of religiously‐based communal activities in the smooth formation of social capital. MRN explains in his qualitative research that this derives from two different sets of religio‐cultural values in the two communities. While the community in the Aceh case study shared its religious belief with that held by the FBO, the community in East Java has its own religio‐cultural interpretation of Islamic belief, which the FBO did not share or try to utilise in its operation.

These findings also speak to the literature on the importance of religious values to social capital, but the literature also suggests a more comprehensive analysis of the circumstances in which faith serves as the basis of social capital formation’ (Candland 2000, p. 355). Candland (2000, p. 371) argues that ‘public policy towards NGOs can influence the ability of these organizations to activate or stimulate social capital for social development’. Aceh's policy towards Islam, which is signified by its implementation of Sharia Law, is certainly conducive to the utilisation of religiously‐oriented communal activity in formulating social capital.

One extra insight provided by the tables, which was not noticed during MRN's qualitative study, is reference to Christianity in the list of words similar to ‘muslim’, which is an indication that both the Islamic FBO and Muslim recipients use this as their religious identity to generate stronger bonding and linking between the FBO and the disaster‐affected community. However, in this list, church (*gereja*), Catholic (*katolik*), and Christian (*nasrani*) appear to be similar words. It seems that when the Muslim identity was formed in the community as well as in the FBO, Christianity was their reference, or perhaps ‘the other’ to help to strengthen their religious identity. This opens up the possibility of making a connection with previously unnoticed elements. Why did the interviewees mention Christianity? Has the community experienced Christian aid organisations before? What might happen if a Christian FBO were to come to this community—can a similar type of smooth formation of social capital happen? These are the questions that MRN's qualitative research has not asked, and it is only via the mixed method that this paper proposes that researchers can go deeper into a hidden element that enriches our understanding of social capital.

In relation to the second question of this paper, the language model validated MRN's original conclusions. The abovementioned relationship between linking and bonding is indicated in his paper. MRN also contends that the existence of an effective village facilitator and religiously‐based communal activities is key. His paper shows how Nopen in the Aceh case study stayed in the village, acquainted himself well with an influential village leader, and mingled with the community. This is comparable to Muhammad in the East Java case study who did not stay in the village unless necessary. This finding can be also confirmed by triangulating with other literature on FBOs—for example, Hirono (2008) underscores the importance of dialogue between outside organisations and a local community by key facilitators. Such dialogue often takes place in local religious scenery. The word embeddings have successfully analysed the interview transcripts and fieldnotes that enhance qualitative research.

Not only does the language model validate research results, but it also adds new and more refined insights to the process of social capital formation. For instance, one insight relates to unpacking the compounding factors that formulate social capital, clarifying more detail, and weighing the relative importance thereof. The language model shows that, of the two factors found in the analysis—the facilitator and religiously‐based communal activities—the former was of utmost importance to community members, owing to their relationship with various stakeholders. The finding was two‐fold. First, the similarity scores of Jodi, Mulyadi, and Nopen in Table 2 show that the effective FBO representative from Jakarta (Jodi) and a local community leader (Mulyadi) are among the top two most significant variables in the perceptions of community members; the village facilitator (Nopen) is the fifth most significant. This is due to their respective connections to various stakeholders, as demonstrated by the similarity scores of these three individuals. Comparing similar individuals in East Java (Saiful, Sarah, and Muhammad), whose similarity scores reveal nothing about stakeholders, the significance becomes clear. Second, Muslim and *gotong royong* (communal work; sharing the burden between the members of a community) are perceived by the villagers as being of third and fourth importance.^8^ Jodi, Mulyadi, and Nopen utilised these to formulate social capital in the community.

## 5 CONCLUSION

This paper began with the same research question as that posed in MRN's paper: ‘How do FBOs conduct their post‐disaster recovery programmes through social capital formation?’. The language model found that Dompet Dhuafa delivered its post‐disaster recovery assistance by building trust, based on key personnel who are well connected to various village stakeholders, and on effective use of religiously‐based communal activities that evoke the norm of togetherness. Such trust building matters to *both* ‘bonding’ and ‘linking’ social capital. Smooth ‘bonding’ within a disaster‐affected community and smooth ‘linking’ with outside organisations connected to each other, while ‘linking’ did not go smoothly when ‘bonding’ within a community was contested. This concurs with the finding of the qualitative research. The AI‐based language model further provides qualitative research with added insights: finer and more systematic specifications regarding the kind of personnel that matter to social capital formation; some extra questions that future qualitative research may explore further, as indicated by the example of Christianity; and clarification of the relationship among the three types of social capital formation.

The methodological implication drawn from the analysis is that the iterative research process of quantitative textual analysis and qualitative contextualisation is a very useful tool to validate ethnologically‐based qualitative research results. It also adds new insights to the making of social capital as one of the ways in which local knowledge manifests itself. The AI‐based language model is particularly useful for the research agenda of locally‐led approaches to disaster response, because it requires a locally‐grounded, context‐specific investigation of local knowledge. The AI‐based language model goes deeper into the contextual meaning of words used by community members. Its research result is far more accurate and far more intricate than could be provided by a publicly available text analysis software based on statistical models.

The above findings, however, have some limitations empirically and methodologically. Empirically, they do not demonstrate whether, and if so how, FBOs deliver post‐disaster recovery assistance by ‘bridging’ with another community. This is because of the different time frame of the datasets used for this paper (covering the period from April to October 2015) and for MRN's research (covering the period from 2015 to 2017). Linking and bonding took place within the project's duration in 2015, but the bridging developed in 2017, after the completion of Dompet Dhuafa's project. One can expect that bridging could have been a spill‐over effect of linking and bonding. But this expectation requires a thorough chronological analysis by extending the period of investigation.

Methodologically, the algorithm chosen for this research (Word2Vec) is well established and widely used, but only allows pair‐wise word/text comparisons. There are algorithms with more capabilities, but at the cost of added complexity of results, making them potentially more difficult to interpret. While an effort was made to make this research as reproducible as possible, the steps to interpret the results from Word2Vec need domain expert oversight (that is, someone who is deeply familiar with the society, culture, and background).

Having said that, this language model analysis has good prospects. Retraining a model is computationally inexpensive and with additional data, models can be updated in the future to provide more detailed insights. Based on the findings of this research, more complex language models can be trained to analyse locally‐led disaster responses.

## ETHICS STATEMENT

This paper reports analysis of primary data. Persons from whom data were collected gave their free, prior, and informed consent. Data have been kept confidential and used anonymously, unless explicit consent was given to reveal their identity.

## Data Availability

Research data are not shared.

## ENDNOTES

^1^
In addition to this question, Muhammad Riza Nurdin's paper asks how assistance helps the affected community to recover, which is beyond the scope of this submission.

^2^
0.77 also fits within the similarity score range used in Jatnika, Bijaksana, and Suryani (2019), in which the word pair ‘coast–shore’ ranges from 0.6140 to 0.8010. They used three vector dimensions (50, 150, and 300) and three window sizes (3, 6, and 9), which are similar to the ones used in our research (300 and 7, respectively).

^3^
There are a few requirements to consider when selecting an algorithm to train a language model. In this research, a few structural conditions, such as data size and single word comparisons, limited the selection of algorithms to Word2Vec, GloVe, fastText, BERT, and similar. The authors also wanted to ensure a generalisable approach and a model with high explicability. The relatively simple architecture of Word2Vec and the word comparisons via a similarity score (cosine similarity) allow results to be explained without additional layers of model complexity.

^4^
To detect different usage of the same word in different regions, we need larger datasets.

^5^
Muhammad and Sarah in Table 1 are pseudonyms, owing to the sensitivity of the East Java case study. In Table 2 (on the Aceh case), real or identifiable nicknames are used because of the person's wish to be named in a publication, or because of prior publication of names on Dompet Dhuafa's website or in online news.

^6^

*Kebersamaan* and *kompak* both mean ‘togetherness’ in different expressions.

^7^
We did not analyse unique keywords that relate to neutral ones such as a location (for example, klangon) and a disaster (for instance, *gempa* (earthquake)). These are unique to location but not to context.

^8^
For an explanation of *gotong yorong*, see Nelson (n.d.).
